# Study of the Electron Beam Melting Process Parameters’ Influence on the Tensile Behavior of 3D Printed Ti6Al4V ELI Alloy in Static and Dynamic Conditions

**DOI:** 10.3390/ma15124217

**Published:** 2022-06-14

**Authors:** Raffaele Barbagallo, Simone Di Bella, Giuseppe Mirone, Guido La Rosa

**Affiliations:** DICAR—Department of Civil Engineering and Architecture, University of Catania, Via Santa Sofia 64, 95123 Catania, Italy; simone.dibella@mtortho.com (S.D.B.); gmirone@dii.unict.it (G.M.); guido.larosa@unict.it (G.L.R.)

**Keywords:** titanium alloy, strain rate, Split Hopkinson Tensile Bar (SHTB), true curve, additive manufacturing

## Abstract

The Ti6Al4V alloy is widely adopted in many high-end applications in different fields, including the aerospace, biomechanics, and automotive sectors. Additive manufacturing extends its range of possible applications but also introduces variations in its mechanical performance, depending on the whole manufacturing process and the related control parameters. This work focuses on the detailed tensile stress–strain characterization at low and high strain rates of a Grade 23 Ti alloy manufactured by electron beam melting (EBM). In particular, the main aim is to study the effect of the variation of the EBM process parameters on the performance of the material and their consequent optimization in order to obtain the best printed material in terms of ductility and strength. The adopted optical experimental setups allow the semi-local scale analysis of the neck section which makes possible the accurate estimation of stress, strain, and strain rate, all over the post-necking range and up to the very incipient specimen failure. Among the EBM printing process parameters, the speed function was previously identified as the one mainly affecting the material performance at static rates. Therefore, two different parameter sets, corresponding to the standard value and to an optimized value of the speed function parameter, respectively, are tested here at dynamic rates of 1, 15, and 700 s^−1^, for assessing the effect of the speed function on the dynamic material response. The results show that the optimized parameter set has a better performance compared to the standard one in terms of strength and ductility. In particular, in both static and dynamic conditions, it presents an increase of the true stress–strain curve (about 5% on average) and an increase of the failure strain (about 11% on average). Moreover, in respect to the standard parameter set, the optimized one is also characterized by a huge increase of the amplification due to the strain rate (about 49% on average for the considered strain rates).

## 1. Introduction

Electron beam melting (EBM), also known by the standardized ASTM 52900 name of electron beam powder bed fusion (EB-PBF), is an additive manufacturing (AM) technique which allows the production of components made of various metallic materials. Recently, AM techniques, and in particular powder bed fusion ones, have found remarkable success in sectors where there is the need to produce components with very complex and high-quality geometry, such as the biomedical and aerospace sectors [1]. Indeed, AM allows for the production of parts with complex geometry that could not be made using traditional production methods [2]. Moreover, the percentage of waste material for each process is significantly reduced, especially in powder bed fusion techniques due to the reuse of the metal powder. EBM has been demonstrated to have some peculiarities with respect to other kinds of AM processes. Similar to some other AM methods, it is a powder bed-based process, but the thermal energy necessary to melt the metal powder is generated by a beam of electrons [3,4]. The process is characterized by a pre-heating stage, which sinters the powder and makes it compact and stable for the following melting phase. The thermal gradient and therefore the internal stresses are reduced by the elevated temperatures during the deposition and pre-melting phases. The high cooling rate of the powder bed, caused by the scanning speed of the electron beam, generates at a microscopic level a finer structure than the same material obtained with traditional techniques. The components made using EBM are built in a vacuum chamber (10^−3^ Pa) which guarantees an environment free of contamination, especially important for those reactive metal alloys such as titanium alloys that have a high affinity to nitrogen and oxygen [5]. The residual stresses, due to the melting phase, are considerably reduced compared to laser printing thanks to the high temperature maintained in the working chamber above 700 °C [6]. The printing procedure requires the continuous repetition of the following four fundamental phases: (1) Deposition of the powder. (2) Preheating of the powder bed in order to compact it and prepare it for the melting phase. (3) Melting the necessary portion of powder through the electron beam. (4) Lowering of the work plane by an amount corresponding to the selected layer thickness value [7].

Recently, the development in the AM field increased the number of printable materials with EBM. Among these materials, titanium alloys play a key role in the aerospace and biomedical industries, especially the Ti6Al4V ELI alloy (Ti grade 23) which is very common in the production of medical devices, due to their lightness and good mechanical properties and corrosion resistance [8,9,10]. Moreover, the absence of toxicity makes them suitable for the realization of implantable medical devices [11,12]. However, the parts made with EBM technology present a high surface roughness; for this reason, especially in the aerospace field, the finished components must be CNC-machined [13]. Ti6Al4V ELI has been studied in the literature [14,15,16] and by companies from several industrial fields. Although, the link between the various printing conditions and the performance of the resulting material is not entirely clear. In fact, extensive data is present in the literature on the mechanical characterization of 3D printed titanium alloys, considering different geometries of the specimens (flat, cylindrical, etc.) and different printing conditions (orientation, as built, machined, heat treated, etc.). However, there is not yet a systematic study on improving the quality of the product nor on optimizing the process parameters. A lot of studies in the literature show materials being characterized based on experimental series using specimens made by the EBM technology with the use of the standard machine parameters that are fixed by the company which produces the printer. With the standard parameters, the printer uses a series of algorithms to continuously adjust the deposition and fusion of the powder. The use of the manual function allows the operator to act on some parameters that control the scanning process and consequently modify the final print result. Some efforts have been made in this direction for the characterization of the 17-4Ph steel produced by selective laser melting [17]. In the case of EBM, the main parameters under study are the speed function, the focus offset, the line offset, and the number of contours. In the case of the ARCAM Q10 EBM printer used in this work, a proprietary automatic algorithm is implemented in the speed function to adjust the scanning speed. The thickness of the melting pool is kept constant by means of this parameter, which acts on the ratio between current and scanning speed. The line offset is the distance between two successive beam lines and its standard value is 0.1 mm [18]. The focus offset controls the size of the spot generated by the beam of electrons. The smaller the spot, the deeper the melting pool is between two successive layers.

This work focuses on the detailed stress–strain characterization at low and high strain rates of a Grade 23 Ti alloy manufactured by electron beam melting (EBM), referring to the semi-local scale of the neck section which allows accurate estimation of stress, strain, and strain rate, extending all over the post-necking range and up to the very incipient specimen failure. In particular, the main aim is to study the effect of the variation of the EBM process parameters on the performance of the material and their consequent optimization in order to obtain the best printed material in terms of ductility and strength. As already highlighted in a previous work of which the main results are reported here for completeness [19], the speed function is the process parameter mainly affecting the material performance at static rates. Therefore, two different parameter sets, respectively corresponding to a standard value and to an optimized value of the speed function parameter, are manufactured and tested here at static and dynamic rates of 1, 10, and 700 s^−1^, for assessing the effect of the speed function parameter on the dynamic material response. As an alternative to the classical elongation-based approach and differing from the various techniques used in the literature to obtain information on the performance of the material from global tensile elongation [20], in this work the experimental procedure will be based on the true stress–true strain data achieved by means of optical measurement of the minimum resistant section area of the specimen [21,22,23,24].

## 2. Materials and Methods

In order to evaluate the influence of the EBM process parameters on the mechanical properties of the Ti6Al4V ELI, static and dynamic tensile experiments on cylindrical specimens have been carried out. An ARCAM Q10 EBM printer was used for the production of the specimens employing titanium alloy powder (Ti6Al4V ELI) with particles ranging between 45 and 150 µm. The thickness of the powder layer was set to 50 µm. The building plate was preheated to a temperature of about 730 °C before the printing process as suggested by the ARCAM standard practice. All the printing jobs were carried out under a controlled vacuum of 10^−3^ mbar in the chamber.

All the specimens have been CNC machined from cylinders of solid material fused through EBM technology, in order to avoid the high surface roughness typical of the additive manufactured products. Moreover, all the samples were built horizontally, i.e., parallel to the production plane, in order to obtain the best repeatability in terms of the mechanical performance of the material. Indeed, in a previous work, it was shown that vertical specimens, in which every cross-section is made by a single printed layer, can generate unreliable results due to the possible presence of *weak layers* which greatly influence the strain localization during the tensile test [19].

**Figure 1:**
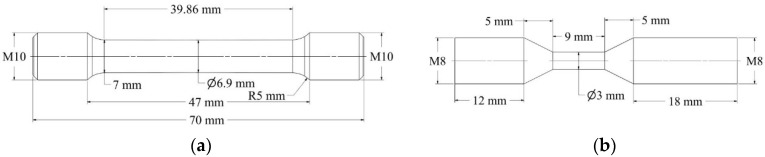
Geometry of the specimens for static (**a**) and dynamic tests (**b**).

Regarding the geometry of the specimens, the authors referred to the ASTM E8 standard related to powder metallurgy. The geometry of the cylindrical specimens for the static tests is shown in Figure 1a. They were machined with ISO M10 threads at both ends for the coupling with the tensile machine. Moreover, they present a slight tapering towards the middle of the gauge length with the diameter of the cross-section varying between 7 mm at the end of the shoulders and 6.9 mm at the central section. The tapering was introduced in order to induce the strain localization in the center of the specimen.

The cylindrical specimens made for the dynamic tests at intermediate and high strain rates were made with the same geometry shown in Figure 1b, with a minimum diameter of 3 mm and a gauge length of 9 mm with no tapering. The specimens were machined with ISO M8 threads at the ends for the coupling with both the SHTB and the hydraulic testing machine.

**Figure 2:**
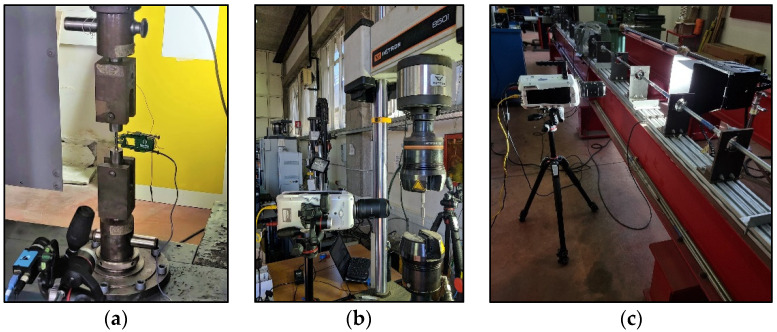
Motor-driven machine and clip gauge used for the static tests (**a**), hydraulic machine used for the dynamic tests at intermediate strain rates (**b**) and SHTB used for the dynamic tests at high strain rates (**c**).

Quasi-static tensile tests were performed with a Zwick/Roell Z100 at a strain rate of 0.003 s^−1^. A standard camera (1280 × 960) was used to monitor the profile of the sample during the test, especially in the post-necking phase. On the other hand, a clip gauge has been used to assess more accurately the elastic properties of the material (Figure 2a).

Dynamic tests at intermediate nominal strain rates of 1 and 15 s^−1^ were carried out with an Instron 8501 hydraulic machine (Figure 2b), while the dynamic tests at high strain rate (700 s^−1^) were carried out with the split Hopkinson tension bar (SHTB) developed at the University of Catania [25] (Figure 2c). All dynamic tests were recorded using a Phantom V711 speed camera. The adopted experimental setups and the corresponding obtained nominal strain rates are recapped in Table 1.

For each tensile test, the image analysis of the recorded video delivered the measurements of the gauge length and of the current minimum cross-section diameter of the specimen at several stages. Then, the engineering curves and the true stress–strain curves were obtained, together with the Young modulus, the ultimate tensile strength, and the maximum elongation values. To obtain the engineering curves, the well-known Equations (1) and (2) have been used [26], in which $\sigma_{eng}$ is the engineering stress, $\varepsilon_{eng}$ is the engineering strain, F is the overall force acting on the specimen, and A and L are the cross-section area and the gauge length of the specimen.

(1) $$\sigma_{eng}=\frac{F}{A_{0}}$$

(2) $$\varepsilon_{eng}=\frac{\Delta L}{L}=\frac{L-L_{0}}{L_{0}}=\frac{L}{L_{0}}-1$$

In the case of the static tests, two different nominal gauge lengths were considered for the mechanical extensometer (10 mm) and for the optical one (30 mm). The mechanical extensometer was used only in the first part of the tests, while the optical one was extended up to failure. In the case of the dynamic tests, a single optical 9 mm gauge length has been considered.

On the other hand, knowing the actual minimum diameter of the specimen, and therefore its minimum cross-section area, it was possible to directly obtain the effective true stress $\sigma_{true}$, true strain $\varepsilon_{true}$, and true strain rate ${\dot{\varepsilon }}_{true}$ for accurately characterizing the material response also at large post-necking strains with the well-known Equations (3)–(5) [26].

(3) $$\sigma_{true}=\frac{F}{A}$$

(4) $$\varepsilon_{true}=2\ln\frac{d_{0}}{d}$$

(5) $${\dot{\varepsilon }}_{true}=\frac{\partial \varepsilon_{true}}{\partial t}$$

Two experimental series were carried out. The first one, already discussed in a previous work [19] and of which the main results are reported below, was focused on the study of the influence of the EBM process parameters (speed function, focus offset, line offset, and number of contours) on the material tensile behavior in static conditions. The chosen parameter sets are shown in Table 2, in which the standard ones suggested by the Arcam company are in the first line. The range of variation of the process parameters was chosen in agreement with the Arcam technicians in order to remain within the security limits of the printer and considering the expected printed material. For each configuration of process parameters, two identical specimens have been made to evaluate the repeatability of the results. The second experimental series focused on the comparison between the performance at intermediate and high strain rates of the standard parameter set and one of the most promising variations found in the first experimental series, i.e., the SF56.

## 3. Results and Discussion

### 3.1. Static Experimental Series

**Figure 3:**
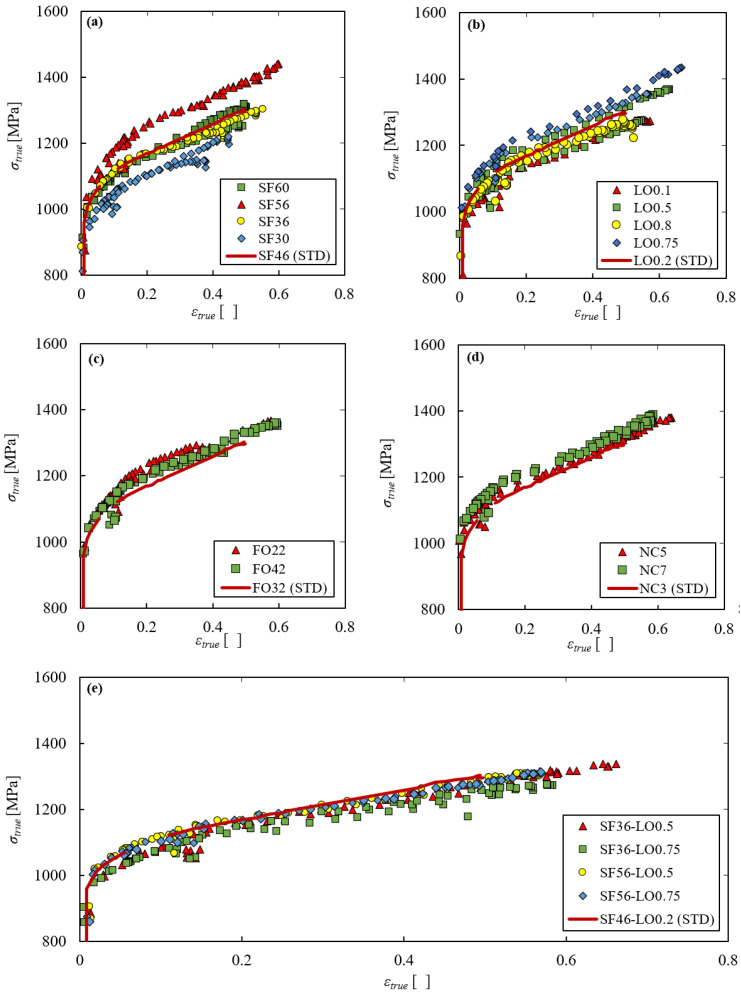
Static experimental series: comparison between the true stress–strain curves obtained with the standard EBM parameter set (solid line) and with the variated ones (scattered lines) changing the speed function (**a**), line offset (**b**), focus offset (**c**), number of contours (**d**) and speed function and line offset (**e**).

**Figure 4:**
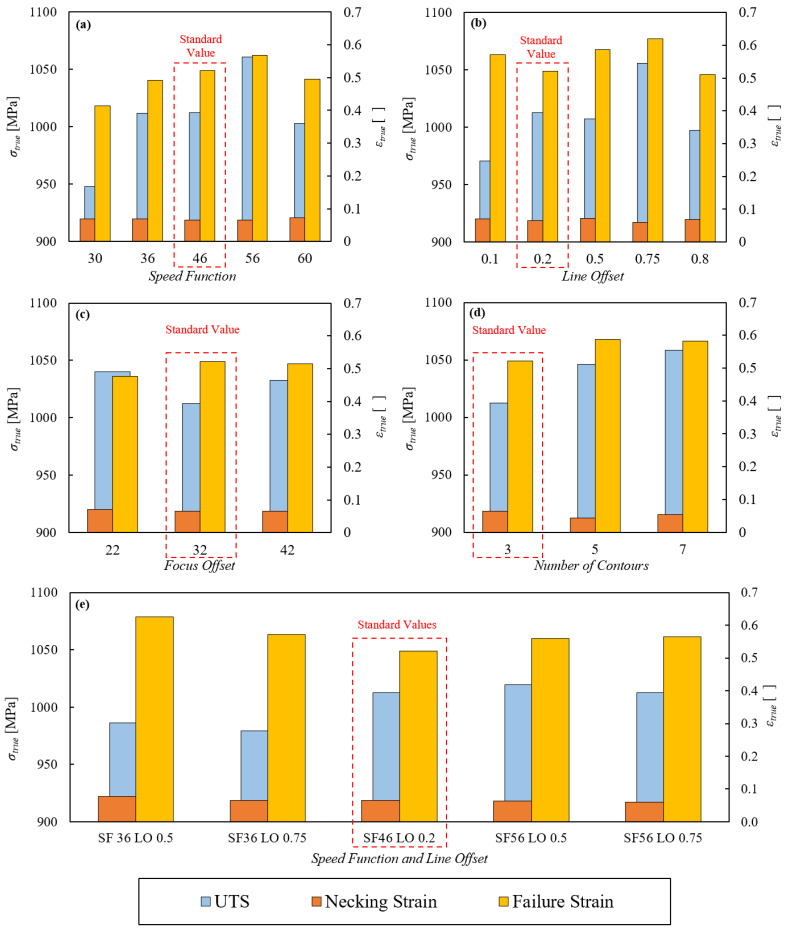
Static experimental series: comparison between the UTS/necking strain/failure strain with the standard EBM parameter set (outlined by the dashed line) and with the variated ones changing the speed function (**a**), line offset (**b**), focus offset (**c**), number of contours (**d**) and speed function and line offset (**e**).

The results of the first static experimental series are recapped in Figure 3 and Figure 4. Figure 3 reports the comparison between the true stress–strain curve obtained with the standard EBM parameter set (solid line) and those obtained with the variated parameters (scatter points). Figure 4 shows the comparison between the ultimate tensile strength/necking strain/failure strain of the standard EBM parameter set (outlined by the dashed line) and of the variated ones.

All the plots in Figure 4 show that the necking strain is substantially not affected by the process parameters, being around 0.06 in all cases. On the other hand, both Figure 3 and Figure 4 clarify that the strength and failure strain are significantly affected by the process parameters. In particular, Figure 3a and Figure 4a consider the analysis of the influence of the speed function parameter, keeping all the other parameters as standard. In this case, it is evident that 56, which is not the standard value, is the optimum value for both strength and ductility. Indeed, higher or lower values of such parameter induce shorter and lower stress–strain curves. Figure 3b and Figure 4b consider the analysis of the influence of the line offset parameter. In this case the optimum value for both strength and ductility is 0.75, once again not the standard one. Figure 3c,d and Figure 4c,d regard the analysis of the influence of the focus offset and number of contours parameters. In both cases, there is no evident difference between the standard curve and the others.

Having found that the most determining parameters are the speed function and the line offset, further tests have been carried out on specimens incorporating both such variated parameters and leaving all others as standard. It turned out that, given the same value of speed function, changing the line offset parameter does not significantly affect the material performance, while changing the speed function leaving the line offset constant has appreciable consequences. The speed function value of 56 is still the optimum one. Therefore, for the successive dynamic experimental series, the SF56 parameter set was chosen to be compared with the standard one in order to verify whether it also shows better performance in terms of ductility and strength in dynamic conditions.

### 3.2. Dynamic Experimental Series

The standard and SF56 parameter sets have been tested at intermediate and high strain rates. The intermediate strain rates of 1 and 15 s^−1^ (D1 and D15 test series) have been achieved by means of an Instron 8501 Hydraulic testing machine while the high strain rate of 700 s^−1^ (D700 test series) has been achieved by means of the SHTB developed at the University of Catania. Thanks to the speed camera acquisitions, in these cases it was also possible to monitor the actual minimum diameter of the specimen during the entire tests. In respect to the static series, this optical approach also provides the possibility of accurately evaluating the effective strain rate developing in the minimum cross-section of the specimen, i.e., the only section which is effectively deformed after the necking onset.

In a typical dynamic test, the strain rate exhibits an initial rise during the transitory time from the zero elongation speed up to the regime value, then it is supposed to remain constant at a plateau until failure because of the constant elongation speed at regime usually imposed. Instead, as shown in previous works by the authors and by other researchers [22,27,28], the effective strain rate spontaneously increases after the necking onset due to the localization phenomena so that, at fracture, the effective strain rate can be ten times greater than the nominal value at the plateau. The typical elongation-based strain measurements with finite gauge lengths only deliver the nominal strain rate history of a test, thereby hiding such phenomena, while the strain measurements based on the evolving diameter correctly highlight the burst of strain rate in the post-necking phase. Therefore, in order to have a complete picture of the effective amplification due to the strain rate, this spontaneous increase of the strain rate must be taken into account.

**Figure 5:**
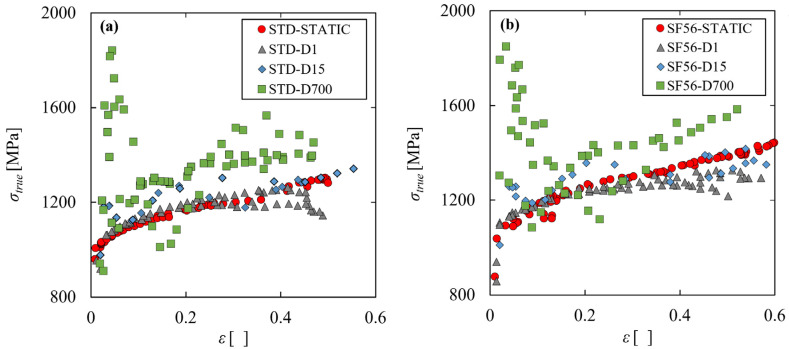
Experimental true stress–strain curves at different strain rates with the standard (**a**) and SF56 (**b**) parameter sets.

Figure 5a,b show the true curves obtained at all the strain rates investigated, with the standard and the SF56 parameter sets, respectively. These figures show that the two parameter sets give a similar behavior. In both cases there is a clear strain rate effect. Indeed, at low strains, the stress–strain curves are roughly ordered according to the corresponding strain rate, with the static curve lower than the D1, D15, and D700. However, at high strains, the dynamic curves tend to decrease, with the D1 and D15 becoming even lower than the static one. This behavior is due to the spontaneous self-heating of the material caused by the fast plastic deformation in quasi-adiabatic conditions. Indeed, the temperature increases with the plastic deformation, causing the thermal softening of the material and the consequent decrease of the curve at high strains.

**Figure 6:**
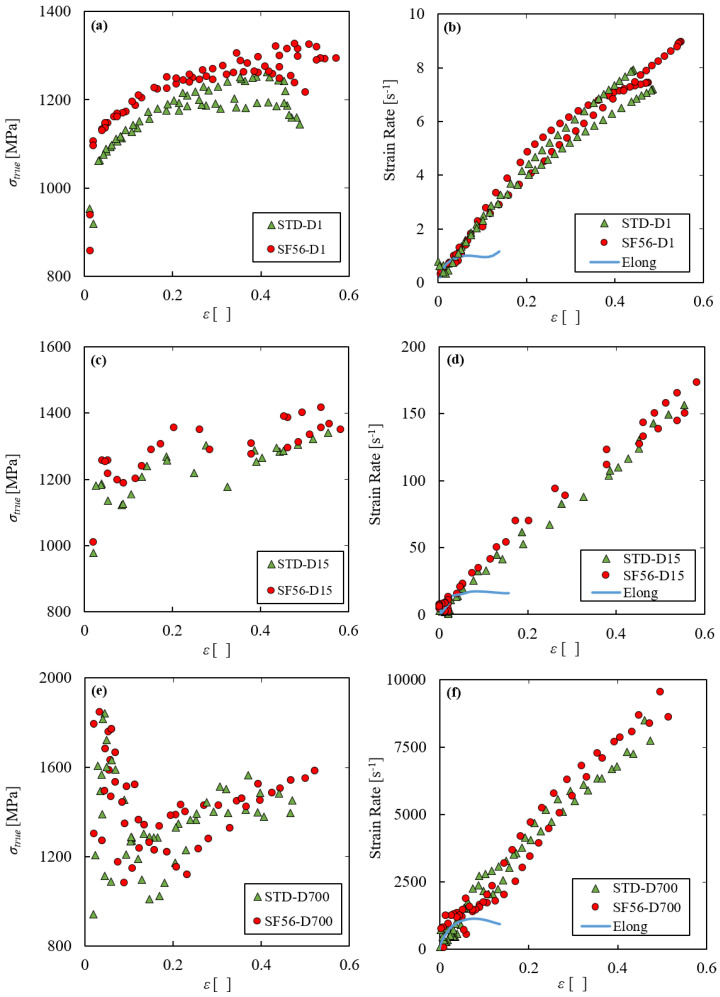
Comparison between the tensile behaviors obtained with the standard and SF56 parameter sets in terms of true stress–strain curves and strain rate vs. strain curves at 1 s^−1^ (**a**,**b**), 15 s^−1^ (**c**,**d**) and 700 s^−1^ (**e**,**f**).

Having ascertained that there is a clear strain rate effect with both the standard and the SF56 parameter sets, it is useful to compare the dynamic mechanical behavior obtained with the two parameter sets at the same nominal strain rate. Figure 6 shows the results of the dynamic tests in terms of true stress–strain curve and strain rate histories, comparing the two parameter sets at the same strain rates of 1 s^−1^ (Figure 6a,b), 15 s^−1^ (Figure 6c,d) and 700 s^−1^ (Figure 6e,f). The displacement imposed by the Hydraulic machine in the cases of the D1 and D15 series and the SHTB input bar preload in the case of the D700 series were identical for the two parameter sets. Figure 6b,d,f show clearly that such equal testing conditions induced the same local true strain rate histories in the specimens in all tests, regardless the parameter set.

Moreover, in the same Figures, one exemplifyingtrue elongation-based strain rate history for each test series is shown as a solid line. It is evident the underestimation of the strain and strain rate in the post-necking phase of the tests of the classical engineering approach. It is essential to highlight that the length-based true stress–true strain data, obtained by the logarithmic transformation of the engineering variables, leave substantially unaltered the degree of underestimation of the simple engineering data. Instead, the effective section-based strain and strain rate at fracture are approximately four and ten times higher than the elongation-based ones, respectively, confirming what the authors shown in [22,27,28]. Given the very low necking strain, the pre-necking plateau of the section-based strain rate curves is very short and not clearly visible for all tests.

In Figure 6 it is possible to note that, similarly at what was already found in the static experimental series, in dynamic conditions the increase of the speed function also gives higher and longer true stress–strain curves, demonstrating better strength and ductility than the standard parameter set. This effect is clearly visible in the D1 and D15 series where the SF56 curves are clearly above the standard ones. Considering the D700 series, the quite significant stress oscillations at the beginning of the tests and the weaker precision give similar curves for all tests.

From the true curves at different strain rates, it is possible to evaluate the dynamic stress amplification R, defined as the ratio between the equivalent stress at a certain strain rate $\sigma_{eq_{D}}$ and the corresponding equivalent stress in static conditions $\sigma_{eq_{ST}}$. As shown in previous works by the authors [22,23] and addressed also by other researchers [29], despite the strain rate varies greatly during a single dynamic test, the stress amplification is not directly related to the actual strain rate. In particular, the authors showed that only the pre-necking plateau value of the strain rate determines the actual behavior of the material. Indeed, after the necking inception, despite the strain rate increases up to ten times its plateau value, the dynamic amplification tends to remain constant. In the case of the material at hand, before the necking onset, the temperature rise due to the self-heating is still low enough to be neglected. Moreover, in the pre-necking phase the true curve of the material is equal to the flow equivalent one. Then, considering the obtained stress values in all dynamic and static tests with the standard and SF56 parameter sets corresponding approximately to the necking strain, it is possible to calculate a value of the dynamic amplification from each dynamic test as shown in Equation (6).

(6) $$R=\frac{\sigma_{eq_{D}}\left(\varepsilon_{neck},\dot{\varepsilon }\right)}{\sigma_{eq_{ST}}\left(\varepsilon_{neck},\dot{\varepsilon }\right)}$$

The ratio of dynamic to static true curves only undergoes moderate variations within the necking onset, so that the ratio in Equation (6) is quite representative of the dynamic stress amplification all over pre-necking strain range.

**Figure 7:**
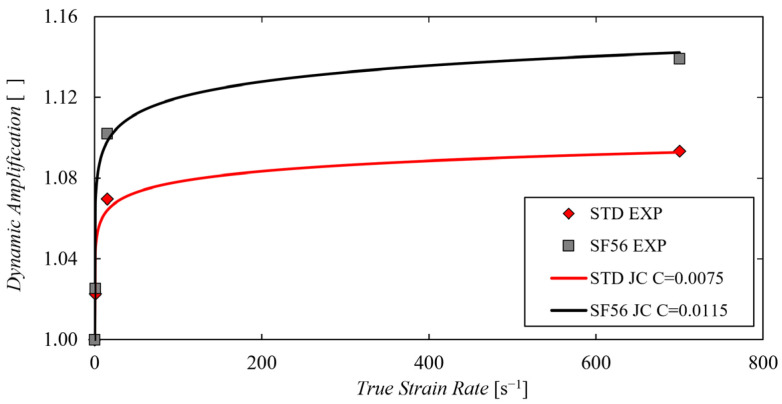
Experimental dynamic amplification obtained with the standard and the SF56 parameter sets (scattered series) and their best approximation considering the Johnson–Cook dynamic term (solid lines).

The values obtained from Equation (6) applied to the dynamic tests are reported in Table 3 and are also plotted against the corresponding strain rate as scattered points in Figure 7. It is evident that, other than having a higher and longer static curve than the standard parameter set, the SF56 demonstrates to have also a much higher strain rate sensitivity, with its *R* values greater than the standard counterparts for all the investigated strain rates. The experimental points of the two parameter sets, have been also best fitted with the classical Johnson–Cook dynamic term function shown in Equation (7).

(7) $$R_{JC}=1+C\ln\frac{\dot{\varepsilon }}{\dot{\varepsilon_{0}}}$$

It was found that the Johnson–Cook dynamic term is able to approximate with reasonable accuracy the obtained experimental points, with the values of the constant *C* of 0.0075 and 0.0115 for the standard and the SF56 parameter sets respectively. These values demonstrate once again the greater strain rate sensitivity of the SF56 parameter set in respect to the standard one.

### 3.3. Discussion

The adopted experimental procedure allowed to accurately describe the tensile mechanical behavior of the Ti alloy at hand, considering different printing parameters and strain rates, in order to identify the most influential process parameters for the mechanical response of the material and their best combination within the security printer limits. Indeed, both the static and the dynamic experiments have been designed with the aim of obtaining the most local material information possible. The utilized optical setups allowed us to monitor the evolution of the minimum cross-section of the cylindrical specimen during the entire tests. By coupling that information with the force readings of the tensile machines, it was possible to directly obtain the true stress–strain curve of the material instead of the classical still widely used engineering curve, much less indicative of the real local behavior of the material. Using such approach, in the first static experimental series it was highlighted that the necking strain is substantially not affected by the process parameters, while strength and failure strain are significantly affected. The *speed function* parameter proved to be the most decisive for the resulting mechanical characteristics of the material. In particular, the SF56 parameter set was found to be the optimum one and it was chosen to be compared to the standard one in dynamic conditions at different strain rates. Analyzing the obtained results, it was possible to clearly highlight that the optimized SF56 parameter set gave better results in both static and dynamic conditions. In particular, Table 4 shows the percentage performance increase from the STD to SF56 parameter set in terms of stress values, failure strain, and dynamic amplification for each test series and on average. The SF56 gave better results in all test series for all the considered characteristics. The biggest improvement regards the stress amplification due to the strain rate (about 49% on average for the considered strain rates), followed by the failure strain (about 11% on average), and by the stress–strain curve height (about 5% on average). Then, the SF56 parameter set does not present any disadvantages with respect to the standard one and represents an overall better choice for whatever application.

## 4. Conclusions

This work focused on the detailed tensile stress–strain characterization in static and dynamic conditions of a Grade 23 Ti alloy manufactured by electron beam melting (EBM). In particular, the main aim was to study the effect of the variation of the EBM process parameters on the performance of the material and their consequent optimization in order to obtain the best printed material in terms of ductility and strength. Thanks to the section-based approach, it was possible to refer to the semi-local scale of the neck section which allows accurate estimation of stress, strain, and strain rate, extending all over the post-necking range and up to the very incipient specimen failure. In particular, the authors studied the effect of the variation of the EBM process parameters on the performance of the material printed with an Arcam Q10 EBM printer.

In a previous work, a static experimental series was carried out and among the EBM printing process parameters, the speed function was identified as the one mainly affecting the material performance at static rates. Then, in this work, a dynamic experimental series (strain rates of 1, 15, and 700 s^−1^) was carried out on cylindrical specimens made with the standard parameter set and with the optimized one identified in the first experimental series for assessing the effect of the speed function parameter also in the dynamic material response. The overall results of the experimental series can be summarized in the following points:

- At static rates, the tensile properties of the Ti alloy at hand are greatly affected by the speed function. Indeed, greater values than the default deliver better strength and ductility until an optimum value at *SF =* 56. Also, the line offset affects the performance of the material, with an optimum value of *LO =* 0.75. On the other hand, changing the focus offset and number of contours values has negligible effects. Considering also the results obtained changing the line offset and speed function values simultaneously, it was shown that the speed function is the most critical parameter regarding the tensile performance of the additive manufactured material. Then, the SF56 parameter set was chosen to be compared to the standard one.
- The standard and the SF56 parameter sets have been tested at 1, 15, and 700 s^−1^. Thanks to the adopted optical techniques, it was shown that the effective section-based strain and strain rate at fracture are approximately four and ten times higher than the elongation-based ones, respectively,
- The results have shown that the optimized SF56 parameter set has better performance compared to the standard one in terms of strength and ductility in both static and dynamic conditions, with higher maximum stress and fracture strain values.
- From the static and dynamic curves, the dynamic amplification obtained with the standard and SF56 parameter sets have been calculated. The results have shown that the optimized parameter set is also characterized by a higher strain rate sensitivity than the standard one.

In conclusion, the optimization of the EBM process parameters gave promising results. The SF56 parameter set demonstrated to give better tensile performance compared to the standard one in both static and dynamic conditions in terms of strength, ductility, and strain rate sensitivity. In particular, in both static and dynamic conditions, it has shown an increase of the true stress–strain curve (about 5% on average) and an increase of the failure strain (about 11% on average). Moreover, in respect to the standard parameter set, the optimized one is also characterized by a huge increase of the amplification due to the strain rate (about 49% on average for the considered strain rates). Future studies will focus on the mechanical behavior of the optimized parameter set under different conditions such as high temperatures and different stress states.

## Figures and Tables

**Table 1:** Experimental setups adopted to obtain the desired strain rates.

Test Series	Experimental Setup	Nominal Strain Rate [s^−1^]
Static	Motor-driven machine Zwick Z100	0.003
Dynamic—Intermediate Strain rates	Hydraulic machine Instron 8501	1
		15
Dynamic—High Strain Rates	SHTB	700

**Table 2:** EBM process parameters sets with the standard values in the first line.

Series	Speed Function	Focus Offset [mA]	Line Offset [mm]	N. of Contours
STD	46	32	0.2	3
SF30	30	32	0.2	3
SF36	36	32	0.2	3
SF56	56	32	0.2	3
SF60	60	32	0.2	3
FO22	46	22	0.2	3
FO42	46	42	0.2	3
LO01	46	32	0.1	3
LO05	46	32	0.5	3
LO075	46	32	0.75	3
LO08	46	32	0.8	3
NC5	46	32	0.2	5
NC7	46	32	0.2	7
SF56_LO075	56	32	0.75	3
SF56_LO05	56	32	0.5	3
SF36_LO075	36	32	0.75	3
SF36_LO05	36	32	0.5	3

**Table 3:** Experimental Dynamic amplification *R*.

Test Series	R STD	R SF56
Static	1	1
D1	1.02	1.03
D15	1.07	1.10
D700	1.09	1.14

**Table 4:** Percentage performance increase from STD to SF56 parameter set in terms of stress values, failure strain, and dynamic amplification.

Performance Increase from STD to SF56			
Test Series	Stress Values	Failure Strain	Dynamic Amplification
Static	7%	9%	-
D1	5%	13%	50%
D15	5%	14%	43%
D700	4%	9%	56%
Average	5%	11%	49%

## Data Availability

Not applicable.
